# Drug‐resistant epilepsy: Drug target hypothesis and beyond the receptors

**DOI:** 10.1002/epi4.12539

**Published:** 2021-10-22

**Authors:** Daniel Fonseca‐Barriendos, Christian Lizette Frías‐Soria, Daniel Pérez‐Pérez, Rosenda Gómez‐López, Dasiel O. Borroto Escuela, Luisa Rocha

**Affiliations:** ^1^ Pharmacobiology Department Center for Research and Advanced Studies México City México; ^2^ Plan of Combined Studies in Medicine (PECEM) Faculty of Medicine UNAM México City Mexico; ^3^ Escuela Nacional de Medicina y Homeopatía Instituto Politécnico Nacional Mexico City México; ^4^ Biomedicum Karolinska Institutet Stockholm Sweden

**Keywords:** antiseizure medications, drug‐resistant, epigenetics, epilepsy, mosaics, oligomers, receptors, target hypothesis

## Abstract

Epilepsy is a chronic neurological disorder that affects more than 50 million people worldwide. Despite a recent introduction of antiseizure drugs for the treatment of epileptic seizures, one‐third of these patients suffer from drug‐resistant epilepsy (DRE). The therapeutic target hypothesis is a cited theory to explain DRE. According to the target hypothesis, the failure to achieve seizure freedom leads to alteration of the structure and/or function of the antiseizure medication (ASM) target. However, this hypothesis fails to explain why patients with DRE do not respond to antiseizure medications of different targets. This review presents different conditions, such as epigenetic mechanisms and protein‐protein interactions that may result in alterations of diverse drug targets using different mechanisms. These novel conditions represent new targets to control DRE.

**Key points**

- Patients with DRE show simultaneous lack of effects of ASMs with different mechanisms.
- Epigenetic changes can lead to simultaneous changes in the expression of different proteins.
- The receptor mosaics expression explains the loss of diverse ASMs efficacy.

## 1 INTRODUCTION

Epilepsy is a common chronic neurological disorder that affects more than 50 million people worldwide.^1^ Antiseizure drug that serves as the first line of treatment is used to control seizures. However, one‐third of patients with epilepsy suffer from drug‐resistant epilepsy (DRE) because they fail to achieve control of seizures despite the appropriate treatment schemes used (in monotherapy or various combinations).^2^

**FIGURE 1:**
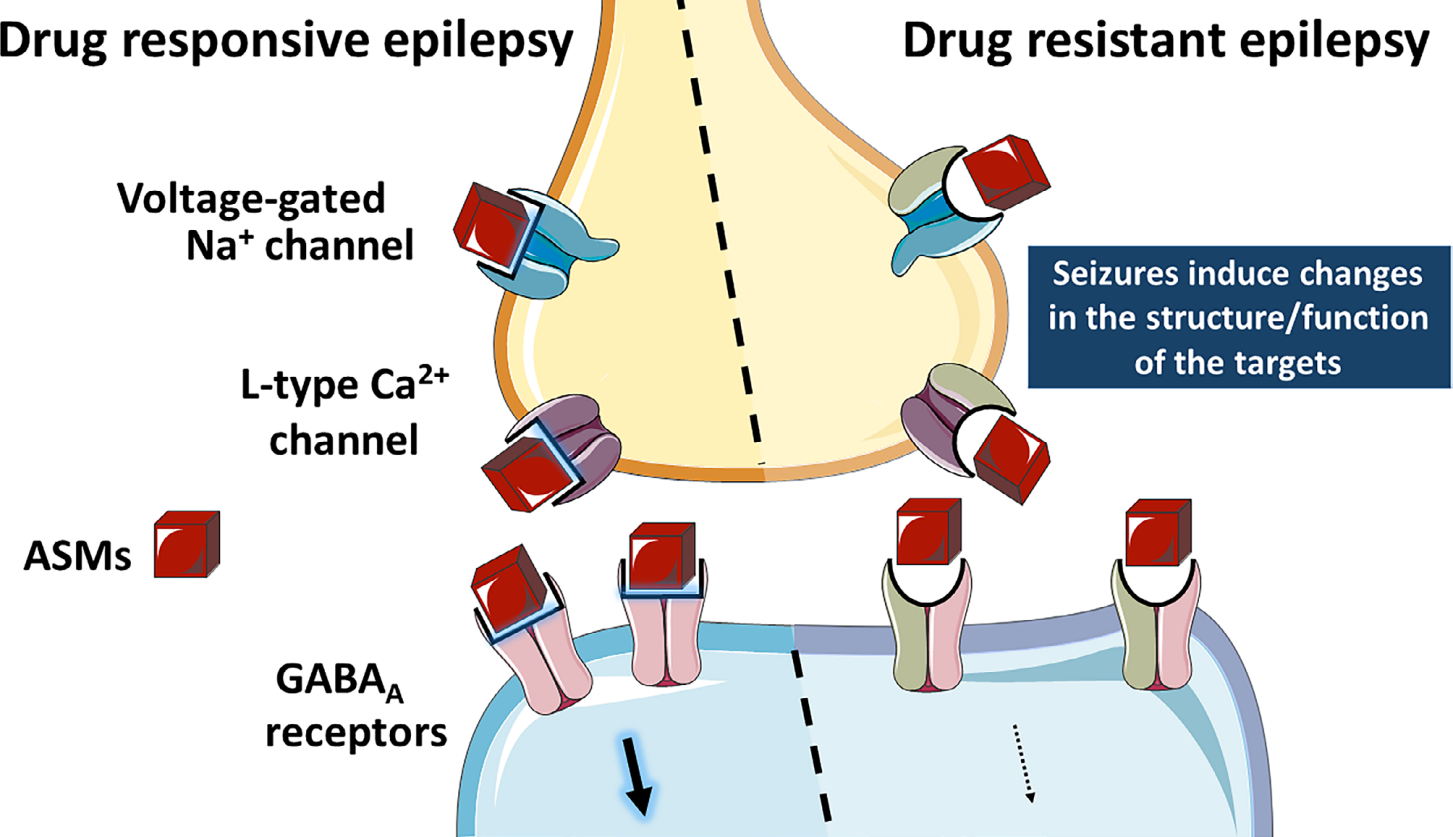
Schematic representation of the antiseizure medication (ASM) target hypothesis. In drug‐responsive epilepsy (DRE) (left side), the ASMs interact with their targets and exert their pharmacological effect. Drug target hypothesis indicates that seizures induce molecular changes alter the structure and/or function of the drug targets. This condition results in the lack of sensitivity to ASMs leading to DRE. Thus, patients with DRE are refractory to several drugs with different mechanisms, which is not explained by the drug target hypothesis. The figure was created using illustrations from Servier Medical Art

According to the target hypothesis that explains the drug‐resistance phenotype in epilepsy, failure to control epileptic activity by antiseizure medication (ASM) results in losing therapeutic efficacy as a consequence of alterations in the structure and/or function of their targets^3^ (Figure 1). In this regard, adopting different therapeutic targets in patients with DRE is crucial, which include alterations in voltage‐gated sodium channels (VGSCs) and γ‐aminobutyric acid (GABA) receptors.

Some studies confirm that resected brain tissue obtained from patients with drug‐resistant temporal lobe epilepsy (DR‐TLE) who have undergone surgery show reduced sensitivity to carbamazepine, a drug that inhibits VGSC.^4^, ^5^ Experimental models have not yet reproduced this condition in DRE. However, studies in models of acute seizures and epilepsy have demonstrated induced alterations in VGSC similar to those detected in brain tissue of patients with the DRE.^6^, ^7^, ^8^

Status epilepticus induced in rats led to increase in spontaneous limbic seizures and window current of VGSC of dentate granule cells. The outcome is associated with a β2 subunit (days 1 and 3 after seizures) and β1 subunit (days 5 and 30 after seizures) reduced expression in VGSC.^8^ From the results, sodium currents were enhanced, leading to aberrant burst discharges.^9^, ^10^ In addition, rats submitted to *status epilepticus* show impaired ASMs efficacy in VGSC, such as phenytoin and lamotrigine. This is more evident when spontaneous seizures are established in animals with a high frequency of ictal events.^11^

GABA_A_ receptor is an important target for several ASMs.^12^ In the hippocampal tissue of patients with DR‐TLE, a decrease in the expression and rearrangement of GABA_A_ receptors subunits has been reported.^13^ Positron emission tomography is an effective neuroimaging method that has increasingly been used for epileptic focus *in vivo* analysis owing to a reduction of GABA_A_ receptor binding and decrease in [^11^C]‐flumazenil binding.^14^, ^15^, ^16^, ^17^ However, in the cortex of patients with DR‐TLE, *in vitro* techniques revealed increased [^3^H]‐flunitrazepam binding.^18^ These findings suggest that the changes in GABA_A_ receptors are region‐specific.

Alterations in expression and/or stoichiometry of α and β subunits of GABA_A_ receptors lead to the loss of efficacy of benzodiazepines and barbiturates in DRE. Studies on animals with spontaneous recurrent seizures previously submitted to *status epilepticus* ^19^ or electrical stimulation^20^ indicate that 40% of the experimental subjects fail to respond to phenobarbital, which is an agonist of GABA_A_ receptor. The issue is associated with decreased expression of GABA_A_ α1‐3,5 and β2/3 receptor subunits in the hippocampus up to 10 weeks after the insult.^21^ An interesting finding is the increased expression of the α4 subunits accompanied by a decrease in the α1 subunit (1‐4 months after pilocarpine‐induced *status epilepticus*), a condition that leads to the lower effectiveness of benzodiazepines.^22^ The increased mRNA expression of α4 and γ2 subunits is reported in the cortex of patients with TLE‐DR.^18^ Thus, the α4βγ2 subtype of GABA_A_ receptors are nonsensitive to benzodiazepine, revealing low brain expression.^23^

Alterations of ASM targets in patients with DRE and experimental models explain the pharmaco‐resistance to individual drugs, that is, drug resistance to GABAergic drugs or VGSC blockers. However, patients with DRE normally fail to respond to diverse ASMs with dissimilar mechanisms, indicating simultaneous changes in different molecular targets. Thus, DRE is associated with a pathological condition that simultaneously modifies diverse targets with different mechanisms. In the subsequent sections of this manuscript, evidence explains epigenetic changes and protein‐protein interactions changes of therapeutic targets with different mechanisms in DRE.

## 2 EPIGENETIC REGULATION

Epigenetics is the series of mechanisms that regulate gene function or expression that are heritable and do not entail a change in DNA sequence.^24^, ^25^ Histone modifications, DNA methylation, noncoding RNAs, and restrictive element‐1 silencing transcription factor/neuron‐restrictive silencing factor (REST/NRSF) are among the epigenetic involved in health and disease.^26^, ^27^

Histone tails and globular domains are the targets of several posttranslational modifications, which include methylation, acetylation, ubiquitination, ADP‐ribosylation, sumoylation, and phosphorylation.^28^ The alterations are associated with active transcription (euchromatin modifications) or inactive regions (heterochromatin modifications).^28^ Previous studies support that epilepsy and seizures induce histone modifications. Brain tissue obtained from patients with DR‐TLE shows upregulation of class 1 histone deacetylase (HDAC) 2, a condition associated with a decrease in histone acetylation and a decrease in gene expression.^29^ Similar results were found in experimental models of seizures. Several histone deacetylases from class 1^29^, ^30^ and class 2^31^, ^32^ are upregulated during the *status epilepticus*–induced epileptogenesis. However, the class 2 member HDAC4 down‐modulates the gene expression of GABA_A_ α1 subunit,^31^ which is benzodiazepine‐sensitive^33^ and the glutamatergic α‐amino‐3‐hydroxy‐5‐methyl‐4‐isoxazolepropionic acid (AMPA) receptor subunit GluA2.^34^ The GluA2 subunit regulates calcium permeability of AMPA receptors,^35^ and expression changes of this subunit is associated with many neurodevelopmental disorders,^36^ tetramerizations,^37^ and neuronal plasticity.^38^ These findings suggest that histone deacetylation following epileptic seizures is associated with simultaneous changes in two different targets: (a) reduced gene expression of α1 subunit of GABA_A_ benzodiazepine sensitive receptors, (b) downregulation of GluA2 subunit of AMPA receptors, a condition that enhances glutamate neurotransmission, aberrant plasticity, and neurotoxicity.^39^

DNA methylation is an epigenetic process of gene silencing mediated by the attachment of methyl groups to cytosine residues, located in promoter regions.^40^

Genome‐wide methylation changes have been evaluated in the brain tissue of patients with DR‐TLE and in epileptic experimental models. A previous study by Miller‐Delaney^41^ indicated that hippocampal resected tissue of patients with DR‐TLE comprised 119 hypermethylated genes and 27 hypomethylated genes, which were compared with the methylation autopsies profile without neurological disorders. Gene ontology revealed that genes with differential methylation were correlated to developmental processes and cellular differentiation, connected to neuronal remodeling and maturation. In addition, neuron‐expressed genes showing hypermethylation in patients with DR‐TLE were associated with subunits of voltage‐dependent calcium channels, voltage‐gated potassium channels, and potassium, inwardly rectifying channels. A major finding of that study was that, despite the intrinsic variability associated with human tissue, less than 150 genes were differentially methylated, which suggested that most of the DNA methylation found in the hippocampus of patients with DR‐TLE was static and disease‐specific.^41^ However, the temporal neocortex of patients with DR‐TLE presents a high expression of DNA methyltransferase 1 (Dnmt1) and Dnmt3a, involved in DNA methylation.^42^

In rats with chronic epilepsy, whole‐genome DNA methylation profiling showed increased DNA methylation in the dorsal hippocampus. However, this analysis fails to show significant changes in the gene expression of the DNA methyltransferases Dnmt1, Dnmt3a, and Dnmt3b in rats with chronic epilepsy.^43^ The results were in contrast with findings obtained from previous studies that focused on the cortex of patients with TLE.^42^ In addition, diverse methylation patterns were detected in hippocampal tissue based on various experimental models of epilepsy (electrical amygdala stimulation, traumatic brain injury, or pilocarpine‐induced *status epilepticus*), ranging from 1121 to 2741 altered regions and hyper‐ or hypomethylation in coding and noncoding sequences.^44^ The present study revealed a broad pathophysiological difference between models. According to the previous information, it is suggest that DNA methylation in epilepsy is disease‐specific that depends on different situations, such as the etiology and chronicity of the disorder, the stage of the disease, the rate of hippocampal sclerosis,^41^, ^45^ and the previous history of prolonged febrile seizures.^46^

In addition, DNA methylation affects several genes, thereby altering protein expression, signaling pathways, neuronal plasticity, inflammation, and immune response, amongst others. Moreover, changes in the transcriptome of different proteins induced by DNA methylation as a consequence of seizures and epilepsy may underlie the resistance of several ASMs.^47^, ^48^

Noncoding RNA is the class of RNA transcripts that are not translated into proteins, but modulate gene expression in a sequence‐specific manner.^49^ Thus, noncoding RNA modulates 30% of all human genes as each noncoding RNA can regulate hundreds of genes.^50^ In hippocampal tissue of patients with DR‐TLE, approximately 18 microRNA (<200 nt) and 4 long noncoding (>200 nt) have been found differentially expressed. In addition, 13 microRNA were methylation‐sensitive, in which DNA methylation exerts strong control of microRNA in DR‐TLE.^41^ Overexpression of miRNA‐134 is in the hippocampal tissue of patients with DR‐TLE and mice sacrificed 3 months after *status epilepticus*.^51^ Previous studies show that miRNA‐134 induces cell damage, has proconvulsant effects, and augments spontaneous seizure activity.^51^ Moreover, microRNA‐134 is negatively associated with synaptic growth and remodeling,^52^ mediated by Limk1 inhibition, which induces dendritic spine growth^53^ and inhibits palmitoyltransferase DHHC9 that regulate protein trafficking^54^, ^55^ to the synaptic membrane in GABAergic neurons.^56^ In addition, microRNA‐155 is upregulated in the hippocampal tissue of patients with DR‐TLE, a condition that correlates with seizure frequency and postsurgical outcome.^57^ The results from an experimental model showed that microRNA‐155 was upregulated up to 60 days after *status epilepticus*, which was when spontaneous seizures were established. In addition, microRNA‐155 provokes cell damage, leads to proconvulsant effects, and increases oxidative stress.^57^ Interestingly, higher microRNA‐155 expression (2.45‐fold) was detected in patients who experienced postoperative seizures, but not in patients without seizures. Computational analysis revealed that microRNA‐155 downregulated the α1 subunit of VGSC expression.^58^ Alterations in this subunit are associated with epileptic pathologies^59^ based on persistent currents, which are shifts in the voltage dependence of activation^60^ and decrease the activity of GABAergic neurons.^61^ These findings demonstrate that epilepsy alters microRNA‐134 and 155 expressions and modifies its progression. In patients with DRE, the absence of response to different ASMs is due to a simultaneous impairment of GABAergic synapse, increasing oxidative stress and altered VGSC as a consequence of microRNAs changes.

REST/NRSF is a protein that acts as a transcriptional repressor. REST binds to RE‐1, a 17‐33 bp sequence found in the DNA, which regulates the expression of approximately 1800 genes.^62^ Once REST binds to RE‐1, it prevents the expression of hundreds of genes and augments the expression of other genes.^63^, ^64^ Hippocampus of patients with DR‐TLE shows REST mRNA overexpression, a finding that positively correlated with seizure frequency.^65^ In addition, REST mRNA overexpression is induced in hippocampal tissue of rats, 24 hours after kainic acid–induced seizures.^66^ The evidence suggests that REST overexpression induced by seizure activity alters many target proteins of the transcriptional repressor, which include brain‐derived neurotrophic factor (BDNF), AMPA receptor GluR2 subunit, and noncoding RNA.^67^ After seizures, REST overexpression may evoke a negative regulatory mechanism and suppress excessive expression of neuronal genes.^66^

Previous studies confirm that failure to achieve seizure freedom in patients with DR‐TLE is associated with the overexpression of REST, which affects multiple target genes, such as expression of VGSC,^68^ calcium channels,^69^ Kv channels,^70^ chloride transporter,^71^ N‐methyl‐D‐aspartate (NMDA) receptor subunits,^72^ µ opioid receptor,^73^ dense‐core vesicles,^74^ and other pre‐ and postsynaptic proteins.

## 3 EPIGENETIC CHANGES INDUCED BY ANTISEIZURE MEDICATIONS

Many ASMs induce epigenetic alterations.^75^ Phenobarbital is associated with a decreased expression of methyltransferases^76^ and increased gene expression. Valproic acid is a histone deacetylase inhibitor^77^ that increases the miRNA‐134 expression in patients with bipolar mania.^78^ Carbamazepine inhibits histone deacetylases^79^ and associates with GABA_A_ gene silencing.^80^ Topiramate is a histone deacetylase inhibitor^81^ that inhibits ethanol‐induced methyltransferase overexpression.^82^ In addition, lacosamide inhibits histone deacetylase.^83^

**FIGURE 2:**
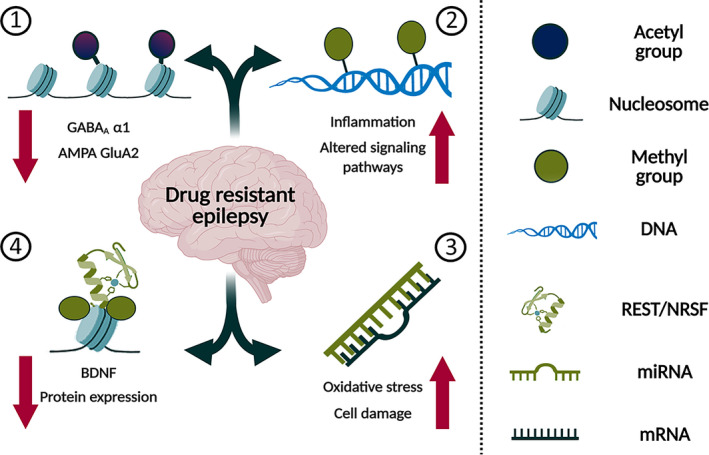
Different epigenetic mechanisms and the drug target hypothesis of drug‐resistant epilepsy. (1) Histone deacetylation downmodulates the gene expression of the benzodiazepine sensitive GABA_A_ α1 subunit and the calcium impermeable AMPA receptor subunit GluA2. (2) Differential DNA methylation has been reported in thousands of coding and noncoding regions, including those associated with protein expression, signaling pathways, inflammation, among others. (3) Noncoding microRNAs overexpression has been associated to cell damage, oxidative stress, and proconvulsant effects, impairing the GABAergic synapse and increasing sodium currents. (4) REST/NRSF overexpression acts as a transcriptional repressor of several target genes including BDNF, ionic channels, glutamatergic receptors, transporters as well as some pre‐ and postsynaptic proteins. The figure was created using illustrations from BioRender.com

The findings raise issues about whether ASM‐induced epigenetic changes aggravate alterations produced by ictogenic activity and contribute to the DRE. Concerning this issue, HDAC inhibitors, such as valproic acid, augment the expression and activity of the multidrug resistance protein 1 transporter in human brain endothelial cells.^84^ The effect facilitates the drug‐resistant phenotype of patients with epilepsy.^85^ On the other hand, specific epigenetic changes were identified in patients with TLE, explaining their resistance to levetiracetam.^86^ Thus, future research is crucial to determine if epigenetic effects induced by ASMs modify epilepsy. Further research is also required to investigate if the expression of the drug‐resistant phenotype depends on clinical factors (dose and duration of the treatment, age of the patient, the condition of the disease, etc). The knowledge derived will help to identify patients with epilepsy susceptible to develop drug‐resistant phenotype when receiving specific ASMs (Figure 2) and will help to design novel epigenetic strategies to control DRE.

## 4 OLIGOMERIC RECEPTOR COMPLEXES AS THERAPEUTIC TARGETS IN DRUG‐RESISTANT EPILEPSY

Some cells require external stimuli to survive. The stimuli are different molecules that include neurotransmitters, hormones, ions, and metabolites, among others. The cell's plasma membrane contains proteins that act as receptors and ion channels used to respond to external signals. Several conditions control the signaling of receptors such as the expression of G proteins, phosphorylation of intracellular residues that terminates receptor‐effector coupling, activation of G protein‐coupled receptor kinases, receptor internalization via clathrin‐dependent endocytosis, etcetera.^87^, ^88^ On the other hand, sustained receptor activation may result in changes in sensitivity, conformation, uncoupling of the effector molecules, and internalization. Several of these receptor regulation mechanisms are based on the drug‐resistant phenotype, and their evaluation has a significant therapeutic implication in designing new strategies to control DRE.

At present, receptors offer physical interaction, representing a cross‐talk mechanism, with relevance to health and disease.^89^ Approximately one‐third of all receptors/ion channels in the cell are oligomers complexes, that is, supramolecular assemblies of two or more associated subunits.^90^ The protein assemblies consist of the same (homo‐oligomers) or different protein subunits (hetero‐oligomers).^91^ For example, G protein–coupled receptors (GPCRs) serve as monomers and homomers (β2‐adrenergic receptors,^92^ the metabotropic glutamate receptor 5,^93^ dopamine D2 receptor,^94^) and heteromers (GABA_B_ hetero‐oligomeric complex^95^), in which allosteric receptor‐receptor interactions modulate the functions of the participating GPCR protomers. In addition, GPCRs form heteroreceptor complexes with ionotropic receptors (D1R‐NMDA)^96^ and receptor tyrosine kinases (FGFR1‐5‐HT_1A_R,^97^ A2AR‐FGFR1^98^), thereby modulating their functions. Moreover, adaptor proteins interact with receptor protomers and modulate their interactions.

Structural‐functional molecular studies show that hetero‐oligomerization between mutant and wild‐type α subunits of the VGSC leads to the channel gating probability impairment.^99^, ^100^ In this channel, the β3 subunit in the VGSC hetero‐oligomer reduces the carbamazepine action.^101^ The hetero‐dimerization of Q2 and Q3 subunits of the voltage‐gated potassium channel increases the sensitivity of the channel for retigabine.^102^ In addition, mutations in the GABA_A_ receptor complex modify the function of the receptors and increase the possibility of forming hetero‐oligomers or homo‐oligomers, thereby facilitating the seizure activity.^103^

However, the expression of some oligomers depends on environmental conditions (pH, temperature, or drug concentration).^104^ The oligomeric complexes' organization and their allosteric protomer‐protomer interactions are reciprocal, highly dynamic, substantially altering the signaling, trafficking, recognition, and pharmacology of the participating protomers. The pattern of changes is unique for each heteroreceptor complex and favors antagonistic or facilitation interaction. Integration of signals to the plasma membrane by homo and heteroreceptor oligomers is crucial. This is based on the hypothesis that learning and memory at the molecular level occur through the reorganization of homo and heteroreceptor complexes in the postsynaptic membrane. Typically, homo and heteroreceptor complexes balance each other, and their disbalance provokes diseases.^105^ In patients with DRE, the waxing‐and‐waning (or fluctuating) pattern in the ASM efficacy^106^ could be associated with the dynamic formation of oligomers.^91^ Future studies are needed to support this hypothesis.

In certain conditions, the receptors and/or ion channels establish physical interactions at a short distance (<40 nm), leading to mosaics (higher‐order oligomers).^107^, ^108^, ^109^ Heteroreceptors are mosaics established with receptors of different pharmacological properties. However, homoreceptors consist of the mosaic that contains different subtypes of the same receptor.^107^, ^110^, ^111^, ^112^, ^113^ Some examples of heteroreceptors are established between κ and δ‐opioid receptors^112^ and the adenosine A1 and dopamine D1 receptors. For example, A2A receptors are activated in homoreceptor complexes.^114^ Thus, the receptor activation in the mosaics alters the function (horizontal molecular network) or the intracellular transductional signals (vertical molecular network) of other receptors.^115^, ^116^ From this, the formation of receptor mosaics explains some clinical features that cannot be explained by the activation of isolated receptors.^117^ Based on the expression of NMDA‐D2 heteroreceptor in the striatum, the activation of D2 receptors can turn glutamatergic‐induced long‐term potentiation into long‐term depression.^118^ In Parkinson's disease, motor alterations are associated with the expression of heteroreceptors of dopamine receptors with adenosine, neurotensin, or different subtype of dopamine receptors.^119^ The different heteroreceptors (serotoninergic and metabotropic glutamate receptors, neurotensin and dopamine receptors, adenosine and dopamine receptors) are associated with the pathophysiology of psychosis and schizophrenia.^120^, ^121^, ^122^ The dopamine D2‐adenosine A2A heteroreceptors lead to the acute locomotor changes and sensitization induced by cocaine.^123^ In addition, the cocaine‐induced psychostimulant is associated with heteroreceptor complex formation between dopamine D2 receptors and NMDA receptor NR2B subunits in the neostriatum.^124^ In patients suffering from depression**,** the neurotrophic and antidepressant effects induced by serotonin agonists are partially mediated by the activation of fibroblast growth factor 1 and 5‐hydroxytryptamine 1A heteroreceptor complexes.^125^

**FIGURE 3:**
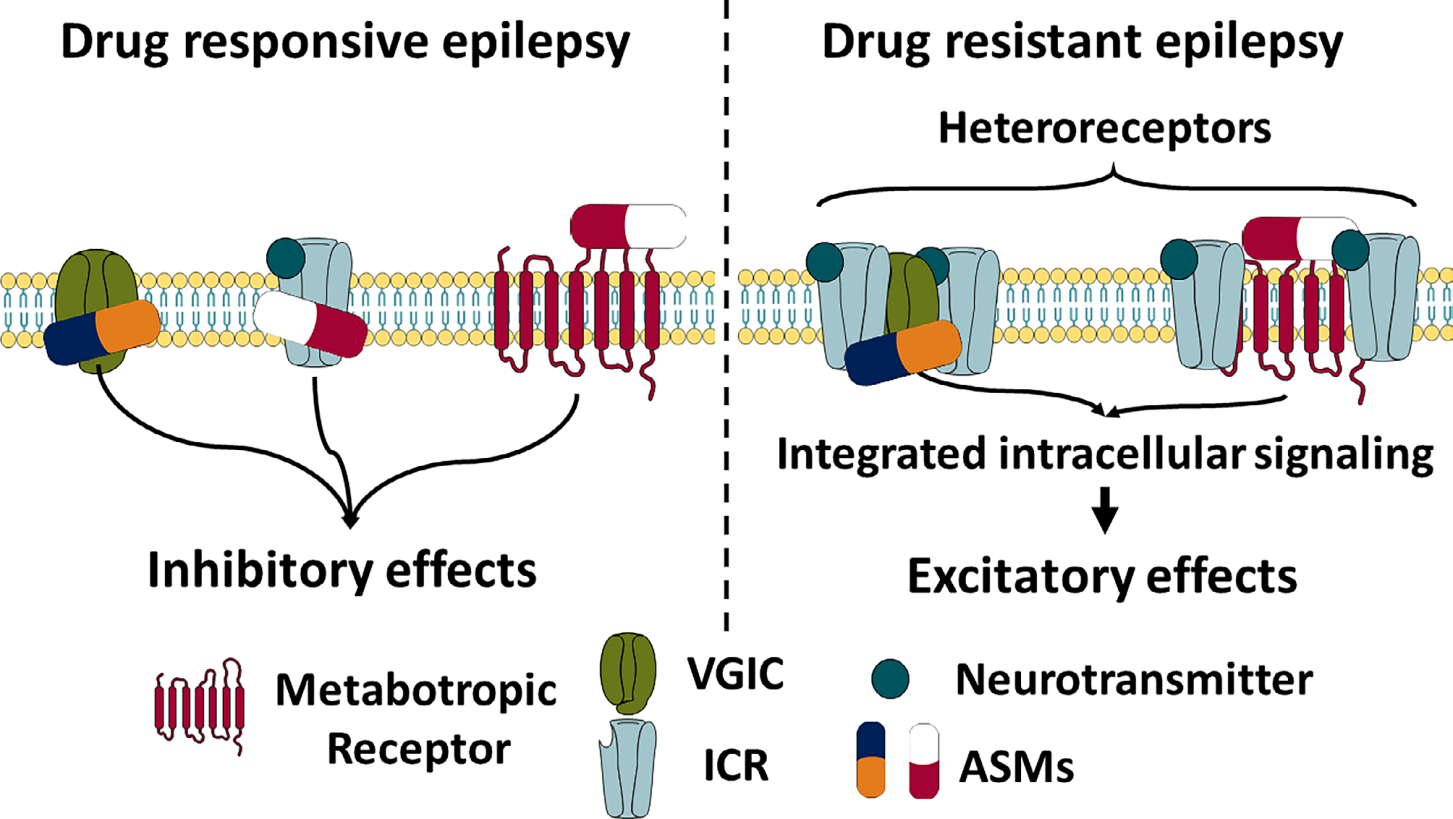
Possible influence of heteroreceptor formation in drug‐resistant epilepsy. In drug‐responsive epilepsy, the antiseizure medications (ASMs) induce inhibitory effects, thus lowering seizure frequency or intensity. On the other hand, the heteroreceptor formation simultaneously changes the intracellular signaling of several receptors with different mechanisms and induces excitatory effects, instead of inhibition. This situation may lead to the drug resistant phenotype of patients with epilepsy. The figure was created using illustrations from Servier Medical Art

At present, information regarding the expression of the receptor mosaics formation associated with epilepsy and DRE is lacking. However, the expression of homo‐ and heterooligomers complexes and receptor mosaics explain the resistance to several ASMs with different mechanisms of action showed by patients with DRE (Figure 3). Thus, patients with DRE have receptor mosaics that are formed by different ASM targets, including the neurotransmitter receptors or voltage‐gated channels. Abnormal interaction within the mosaics could modify their sensitivity to diverse ASMs, thereby explaining the multidrug resistance phenotype. Everitt^126^ hypothesized that a pathological memory, called drug memory, is associated with drug addiction. They also proposed that its molecular basis could lead to novel drug abuse user therapies. Our hypothesis on learning and memory molecular basis^127^ is in line with the Everitt hypothesis. We suggest that drug memories can be produced through a reorganization of the homo and heterooligomeric complexes in synapses and extra‐synaptic regions.

Future studies should determine if the DRE condition is associated with the dynamic expression of receptor mosaics. In addition, the abnormal receptor‐receptor interactions in DRE opens the novel therapeutic approaches^117^ depending on the heteroreceptor or mosaic expression.^128^, ^129^

## 5 CONCLUSION

The DRE target hypothesis indicated that the loss of therapeutic efficacy of ASMs was due to alterations in the molecular target. However, patients with DRE suffer from the loss of therapeutic efficacy of ASMs with dissimilar mechanisms. Other mechanisms can induce simultaneous changes in different molecular targets. In this review, information obtained from the literature suggests that altered gene expression due to epigenetic regulation and receptor mosaics formation is involved in the loss of several targets efficacy.

An important issue is to consider how the brain of patients with epilepsy is organized and the concept of the epileptic focus. The surgical strategy focuses to reduce seizure activity through resection of the epileptogenic region (the brain area sufficient for the generation of spontaneous seizures) and the distant irritative zone (characterized by spontaneous interictal spike activity, and involved in the seizure propagation).^130^, ^131^ Eventually, the irritative zone zone serves as a control condition, compared with the epileptogenic region of the same patient. However, studies indicate that both regions present significant changes, suggesting an increased excitatory influence in the epileptic focus and decreased inhibitory neurotransmission in the irritative zone.^132^ Indeed, the irritative zone increases vascularity, microlesions, and marked activation of mitogen‐activated protein kinase and cAMP‐response element‐binding protein (MAPK/CREB, a signaling pathway involved in synaptic plasticity).^133^ Previous studies that focused on the human brain with epilepsy revealed a high expression of long noncoding RNA (lncRNAs) genes involved in synaptic plasticity, a situation that correlated with the interictal spiking activity.^134^ From this information and considering that lncRNAs involve in gene regulation,^135^ the epileptogenic region and irritative zone present different patterns of gene and protein expression. Thus, the conclusions about changes in targets obtained from the evaluation of epileptogenic region vs irritative zone could lead to misconceptions. Future studies should identify changes in targets and mechanisms in the different zones of the epileptogenic region.

## CONFLICT OF INTEREST

No authors disclose conflict of interest. The authors confirm that they have read the Journal's position regarding the ethical publication and affirm that this report is consistent with those guidelines.
